# Dynamic Transcriptome Analysis Reveals Uncharacterized Complex Regulatory Pathway Underlying Dose IBA-Induced Embryogenic Redifferentiation in Cotton

**DOI:** 10.3390/ijms21020426

**Published:** 2020-01-09

**Authors:** Yupeng Fan, Xiaoman Yu, Huihui Guo, Junmei Wei, Haixia Guo, Li Zhang, Fanchang Zeng

**Affiliations:** 1 State Key Laboratory of Crop Biology, College of Agronomy, Shandong Agricultural University, Tai’an 271018, China; fanyupeng@chnu.edu.cn (Y.F.); 15890636826@163.com (X.Y.); hhguo@sdau.edu.cn (H.G.); nxzkwjm@163.com (J.W.); diya_haixiaguo@163.com (H.G.); 15610418001@163.com (L.Z.); 2 College of Life Sciences, Huaibei Normal University, Huaibei 235000, China

**Keywords:** cotton, somatic embryogenesis, embryogenic redifferentiation, indole-3-butyric acid (IBA), embryogenic inductive effect, IBA dose effect, molecular signaling and regulation pathway

## Abstract

The somatic embryogenesis (SE) process of plants is regulated by exogenous hormones. During the SE, different genes sensitively respond to hormone signals through complex regulatory networks to exhibit plant totipotency. When cultured in indole-3-butyric acid (IBA) concentration gradient medium supplemented with 0 mg dm^−3^, 0.025 mg dm^−3^, and 0.05 mg dm^−3^ IBA, the callus differentiation rate first increased then decreased in cotton. To characterize the molecular basis of IBA-induced regulating SE, transcriptome analysis was conducted on embryogenic redifferentiation. Upon the examination of the IBA’s embryogenic inductive effect, it was revealed that pathways related to plant hormone signal transduction and alcohol degradation were significantly enriched in the embryogenic responsive stage (5 days). The photosynthesis, alcohol metabolism and cell cycle pathways were specifically regulated in the pre-embryonic initial period (20 days). Upon the effect of the IBA dose, in the embryogenic responsive stage (5 days), the metabolism of xenobiotics by the cytochrome P450 pathway and secondary metabolism pathways of steroid, flavonoid, and anthocyanin biosynthesis were significantly enriched. The phenylpropanoid, brassinosteroid, and anthocyanin biosynthesis pathways were specifically associated in the pre-embryonic initial period (20 days). At different developmental stages of embryogenic induction, photosynthesis, flavonoid biosynthesis, phenylpropanoid biosynthesis, mitogen-activated protein kinase (MAPK) signaling, xenobiotics metabolism by cytochrome P450, and brassinosteroid biosynthesis pathways were enriched at low a IBA concentration. Meanwhile, at high IBA concentration, the carbon metabolism, alcohol degradation, circadian rhythm and biosynthesis of amino acids pathways were significantly enriched. The results reveal that complex regulating pathways participate in the process of IBA-induced redifferentiation in cotton somatic embryogenesis. In addition, collections of potential essential signaling and regulatory genes responsible for dose IBA-induced efficient embryogenic redifferentiation were identified. Quantitative real-time PCR (qRT-PCR) was performed on the candidate genes with different expression patterns, and the results are basically consistent with the RNA-seq data. The results suggest that the complicated and concerted IBA-induced mechanisms involving multiple cellular pathways are responsible for dose-dependent plant growth regulator-induced SE. This report represents a systematic study and provides new insight into molecular signaling and regulatory basis underlying the process of dose IBA-induced embryogenic redifferentiation during SE.

## 1. Introduction

Somatic embryogenesis (SE) is a process by which embryogenic differentiation is generated directly from somatic cells, similarly to the development of zygotic embryos, and it involves the developmental reprogramming of somatic cells toward the embryogenesis pathway. Somatic embryogenesis can be carried out in vitro under artificially controlled conditions to generate the most complete cell totipotency. Somatic embryogenesis plays an important role in cell fusion, genetic engineering, and somaclonal variation. SE was discovered in the 1950s [1]. Since then, scientists have conducted extensive research on the mechanism of somatic embryogenesis. It has been concluded that under artificially controlled in vitro conditions, plant somatic cells can regain their ability to regenerate after cell dedifferentiation and redifferentiation and can develop into seedlings. At present, more than 100 species are known to be capable of plant regeneration through somatic embryogenesis, mainly including alfalfa, soybean, cotton, spruce, pine and cypress. However, few of the molecular events involved in the transition of a somatic cell to an embryogenic-competent cell are known thus far [2,3].

Cotton is globally one of the most important commercial crops. Biotechnology approaches, including genetic engineering and tissue culture, have been widely applied to cotton breeding. All transgenic cotton production demands a productive plant regeneration procedure from the somatic cells in cotton. Cotton transgenic technology relies heavily on somatic embryogenesis [4]. Genotypes have a decisive influence on somatic embryogenesis in cotton. Xie et al. [5] believed that the genotype of the material is the first factor to affect the somatic embryogenesis and plant regeneration of cotton, followed by the type of phytohormone and its concentration. Since the 1970s, more than 100 cotton varieties have been used for tissue culture research. Approximately one half of these materials have embryogenic ability, while the remaining half are unable or extremely unlikely to induce somatic embryos [6,7]. Most cultivars have been recalcitrant, and few reports of high-frequency regeneration in cotton via SE have come forth, due to a genotype-dependent response [8,9,10].

Exogenous hormones are another important factor that induce somatic embryogenesis in cotton in addition to genotype. Currently, 2, 4-dichlorophenoxyacetic acid (2, 4-D), indole-3-butyric acid (IBA) and kinetin (KT) are commonly used. Some scholars have pointed out that among all auxins and auxin analogues, 2, 4-D has a relatively good effect on promoting the induction of explant callus [8,11,12,13]. Furthermore, the addition of 2, 4-D in a suspension culture contributes to the formation of somatic embryos [14]. It has been reported that the combination of IBA and KT hormones contributes to embryogenic callus (EC) differentiation [12,15,16,17]. Moreover, the combination of IBA and KT has good effects on some stubborn varieties in which induced differentiation has been difficult. Zhu et al. [15] found that simultaneous treatment with 2, 4-D, indole acetic acid (IAA) and KT yielded better effects, but the calli induced by the 2, 4-D medium must be transformed to 2, 4-D removed or replaced medium to ultimately undergo somatic embryogenesis. If 2, 4-D was removed and the hypocotyl segment was inoculated with ZSW-2 (Murashige and Skoog (MS) + 0.1 mg dm^−3^ KT + 0.1–0.5 mg dm^−3^ IBA + 3% (*w*/*v*) glucose + 6.5 g dm^−3^ agar, pH 6.2) [15] medium containing only KT and IBA, the ideal state of callus was achieved, and, therefore, the appropriate ratio of cytokinin and auxin yields a better induction rate. Chen et al. [18] found that an MSB (MS inorganic salt + B5 vitamin) medium supplemented with IBA could directly induce embryogenic calli and that appropriate concentrations of KT could promote the effect of IBA. IBA is used to induce root organogenesis [19], but studies have found that either IBA alone or in combination with KT can directly induce a certain amount of embryogenic callus from the hypocotyl segment. More extensive experiments are yet to be done to determine the mechanism through which IBA acts.

Furthermore, the auxin and cytokinin signaling pathways play an important regulatory role in cotton somatic cell dedifferentiation and embryogenic cell redifferentiation [20,21,22,23]. Cotton plantlets can be regenerated via SE using various combinations of plant growth regulators, such as 2, 4-dichlorophenoxyacetic acid (2, 4-D), indole-3-butyricacid (IBA), and naphthalene acetic acid (NAA) in combination with Kinetin (KT) [24]. Global transcriptome analyses have suggested that the auxin and cytokinin signaling pathways are critical in the dedifferentiation of somatic cells and the redifferentiation of SEs in cotton. Additionally, stress-responsive genes and pathways have also been found to be involved in SE [21]. The ethylene response factor (*ERF*) plays an important role in hormone signal transduction and interconnecting different hormone pathways [25]. Despite these reports, we still know little about the molecular mechanisms underlying plant growth regulator-induced SE in cotton.

Scientists found that auxin plays an important role in this process [21] while using small RNA sequencing (sRNA-seq) and degradome sequencing to find multiple small RNAs and their targets in somatic embryos. The occurrence of differential expression [26] indicates that cotton somatic embryogenesis is regulated by complex gene expression. With the development of next-generation sequencing technology and the continuous improvement of cotton genome information [27,28], more than 5000 differential expression genes have been discovered in cotton somatic embryogenesis using digital gene expression (DGE) technology. Our primary strategy was to use transcriptome sequencing techniques with the YZ-1 genotype material of highly embryogenic to identify genes expressed during early induction of SE under induction culture treatment in the IBA concentration gradient medium.

## 2. Results

### 2.1. Callus Differentiation Rate of Cotton Induced by Different IBA Concentration and KT Combinations

Hypocotyl segments 5–7 mm in size were cultured on an MS medium supplemented with 0.1 mg dm^−3^ 2, 4-D and 0.1 mg dm^−3^ KT (this hormone combination is named C3 in the following description). After 6 weeks of culture, all explants were transferred to different IBA concentration gradient mediums to induce embryogenic callus. The rate of embryogenic callus induction was calculated after 30 days. The results show that the callus tissue differentiation rate was the lowest on the medium supplemented with 0.1 mg dm^−3^ KT alone. After adding different IBA based on the 0.1 mg dm^−3^ KT, the callus tissue differentiation rate increased significantly, especially in the 0.1 mg dm^−3^ KT + 0.025 mg dm^−3^ IBA medium (Table 1). The use of KT and IBA in the differentiation process of cotton calli is beneficial.

### 2.2. Density Distribution of Reads on Chromosomes

**Figure A1:**
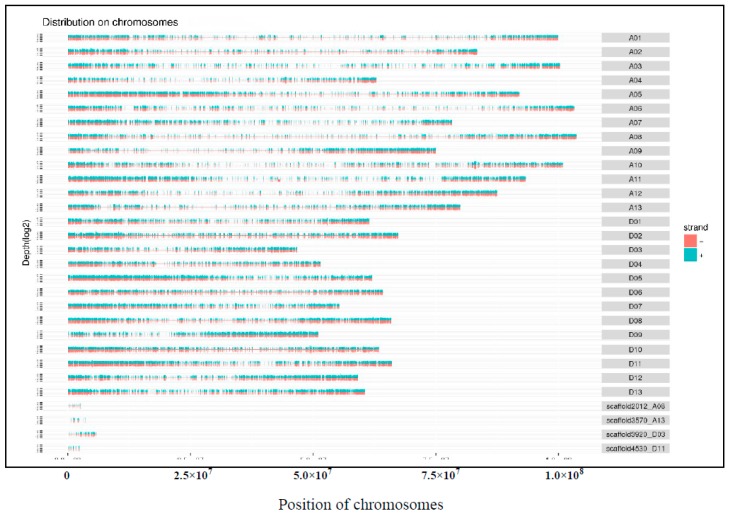
Density distribution map of reads on chromosomes. Ordinate is the depth distribution of the sequence on the chromosome, and log2 value is taken. Abscissa is the length of the chromosome. (A01–A13 refers to chromosome 01–13 in cotton A genome, and D01-D13 refers to chromosome 01–13 in cotton D genome).

The total reads were mapped to all of the chromosomes in the genome, as shown in Figure A1. The specific mapping method was used to calculate the number of reads aligned to the base position in the window, calculate its depth distribution on the chromosome, and take the log2 value. Under optimal conditions, the longer the length of the entire chromosome, the more reads localized within the chromosome. From the diagram of the relationship between the number of reads localized in the chromosome and the length of chromosome, the uniformity of sequencing can be seen more intuitively.

### 2.3. Screening of Differentially Expressed Genes

The Reads Per Kilo bases per Million reads (RPKM) method was used to calculate the gene expression level. The sequencing depth and gene length were also normalized, making the gene expression levels estimated for different lengths of genes at different sequencing depths comparable.

**Figure 1:**
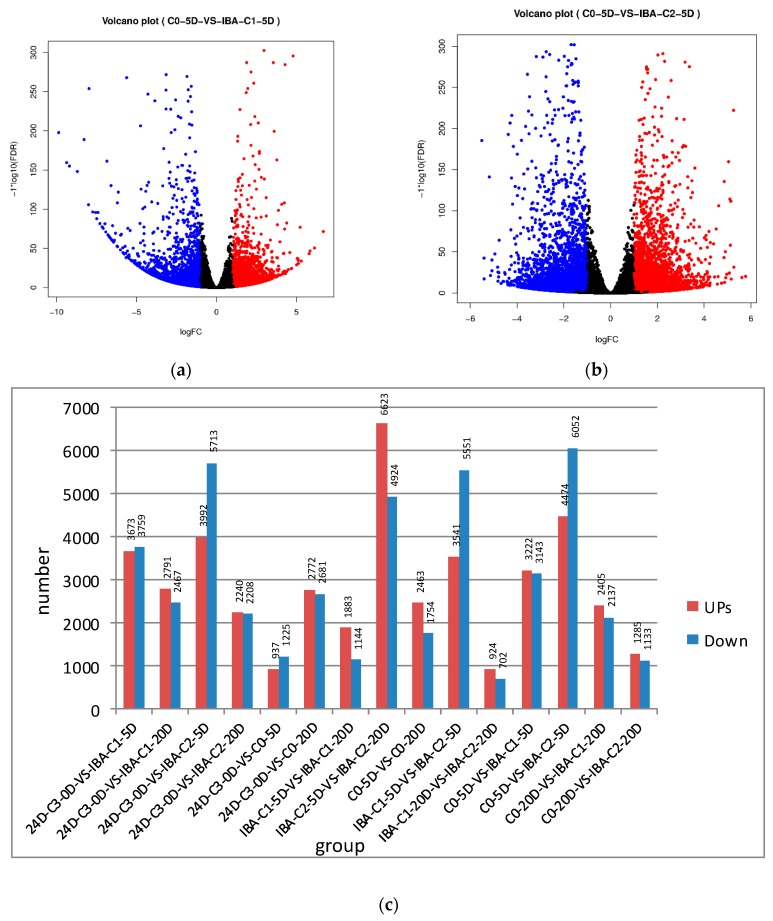
The up- and down-regulation of gene expression between the comparison group. (**a**,**b**) The volcano plot of differentially expressed genes. (The red dots represent up-regulated genes, the blue dots represent down-regulated genes, the abscissa represents the expression multiple change of genes in different samples, and the ordinate represents the statistical significance of the difference in gene expression) (**c**) The up- and down-regulation of gene expression was compared between samples.

Volcano plots were drawn based on the results of the tests and were screened according to the differential significance criteria (greater than two-fold changes in the expression of differential genes and FDR ≤ 0.05), and the statistically significant differences in gene expression were measured (Figure 1). The volcano plot indicates that there are many genes up- and down-regulated in callus tissue compared to the C0 treatment group (Figure 1a,b).

### 2.4. Overall Transcriptome Sequencing Analysis

In the process of cotton somatic embryogenesis, transcriptome sequencing analysis was used by high-throughput sequencing technology. Comparing with the database, 1542–11,235 genes were matched to the Non-Redundant Protein Sequence Database in GenBank (Nr), 1038–7564 genes were matched to the Gene Ontology (GO) database, and 333–2828 genes were matched to the Kyoto Encyclopedia of Genes and Genomes (KEGG) database (Table 2).

### 2.5. Screening of Differentially Expressed Genes from Venn Diagram between Different Comparison Groups

**Figure 2:**
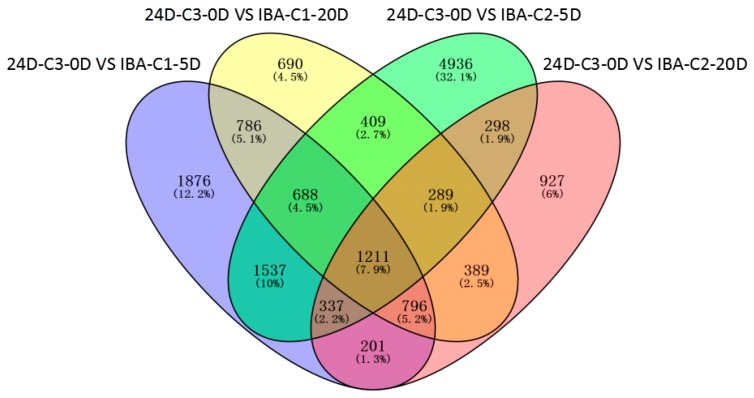
Venn diagram of differentially expressed genes between different comparison groups.

Transcriptome analysis found 1211 common genes expressed during 2, 4-D-induced dedifferentiaion (24D-C3-0D) compared with series of IBA-induced redifferentiaion (Figure 2). In the embryogenic responsive stage of redifferentiation, the higher IBA concentration (24D-C3-0D VS IBA-C2-5D) induced much more specific expression genes (4936) compared with 1876 genes in the lower IBA (24D-C3-0D VS IBA-C1-5D) (Figure 2), but the callus differentiation rate was low (52.63% VS 72.22%) (Table 1). The results suggest that excessive expression genes are activated in the higher concentration, but this is not beneficial to embryogenic responsiveness for redifferentiation (5 days). An appropriate and concerted amount of gene expression facilitates differentiation. As in the pre-embryonic initial period (20 days), the callus could adapt to different IBA concentrations—therefore, the specific expressed genes have little difference post responsive stage.

### 2.6. Type and Number of Alternative Splicing

**Figure 3:**
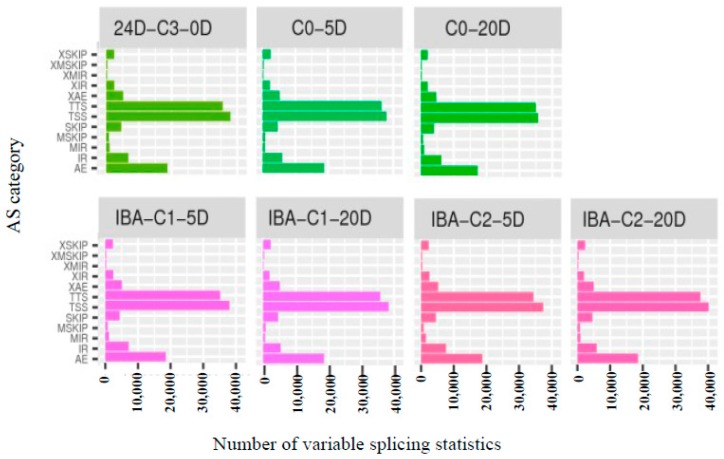
Type and number of variable splicing statistics. AE: Alternative exon ends; IR: Intron retention; MIR: Multi-IR; MSKIP: Multi-exon SKIP; SKIP: Skipped exon; TSS: Alternative 5′ first exon; TTS: Alternative 3′ last exon; XAE: Approximate AE; XIR: Approximate IR; XMIR: Approximate MIR; XMSKIP: Approximate MSKIP; XSKIP: Approximate SKIP.

Variable splicing allows a gene to produce multiple mRNA transcripts, and different mRNAs may be translated into different proteins. Therefore, multiple proteins can be produced by the variable splicing of a gene, which greatly increases protein diversity. ASprofile software (http://ccb.jhu.edu/software/ASprofile/, v1.0.4, The Center for Computational Biology at Johns Hopkins University, Baltimore, MD, USA) was used to classify and count the variable splicing events of StringTie (v1.0.4)-predicted transcripts, as shown in Figure 3. There were many variable splicing events in all samples. The results show that the overall patterns of variable splicing events were similar among all samples, with more than 66% of the variable splicing events being concentrated in the TTS (Alternative 3′ last exon), TSS (Alternative 5′ first exon), and AE (Alternative exon ends (5′ 3′, or both) categories. The results indicate that there is no difference between the samples treated with different IBA concentrations. Variable splicing events proceed steadily and are not affected by different IBA concentrations.

### 2.7. Functional Annotation of Differentially Expressed Genes

**Figure A2:**
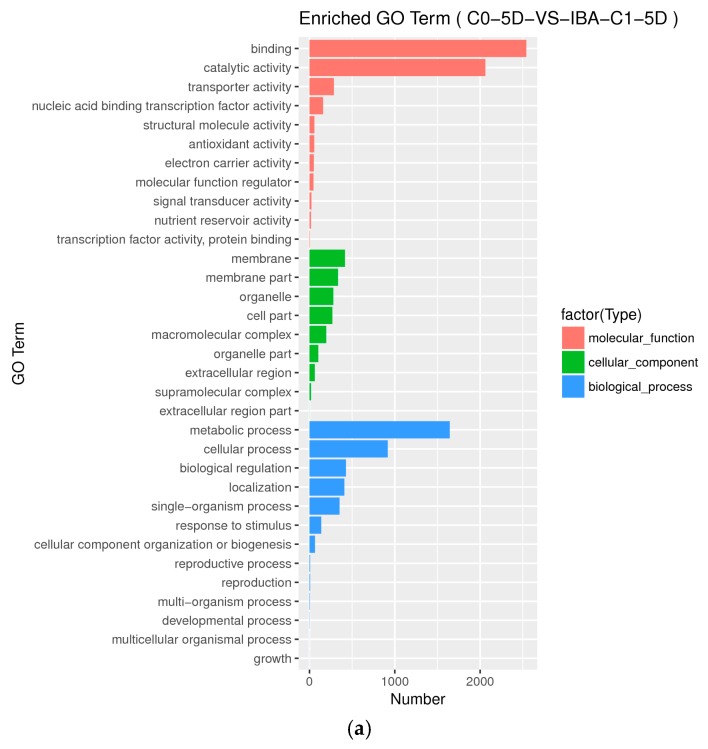

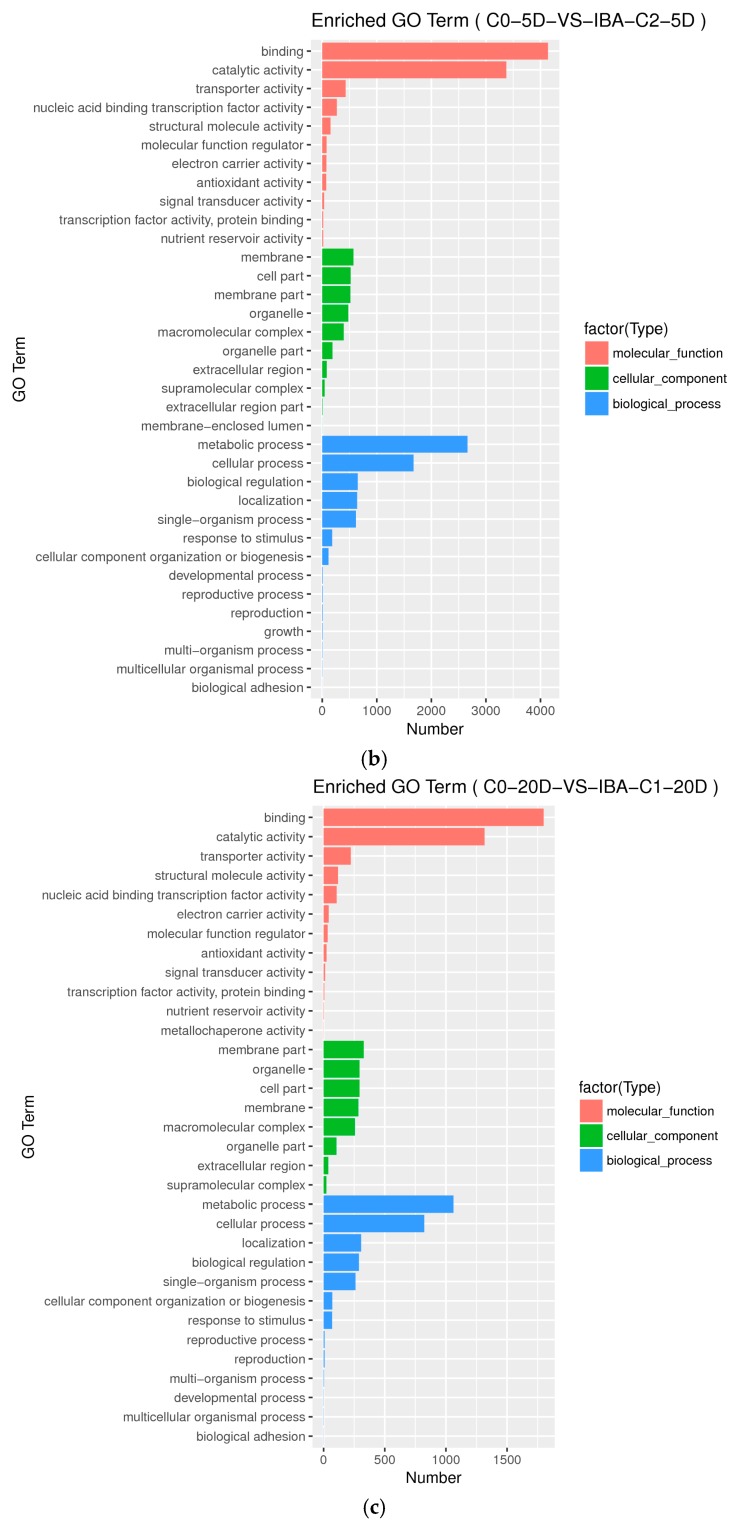

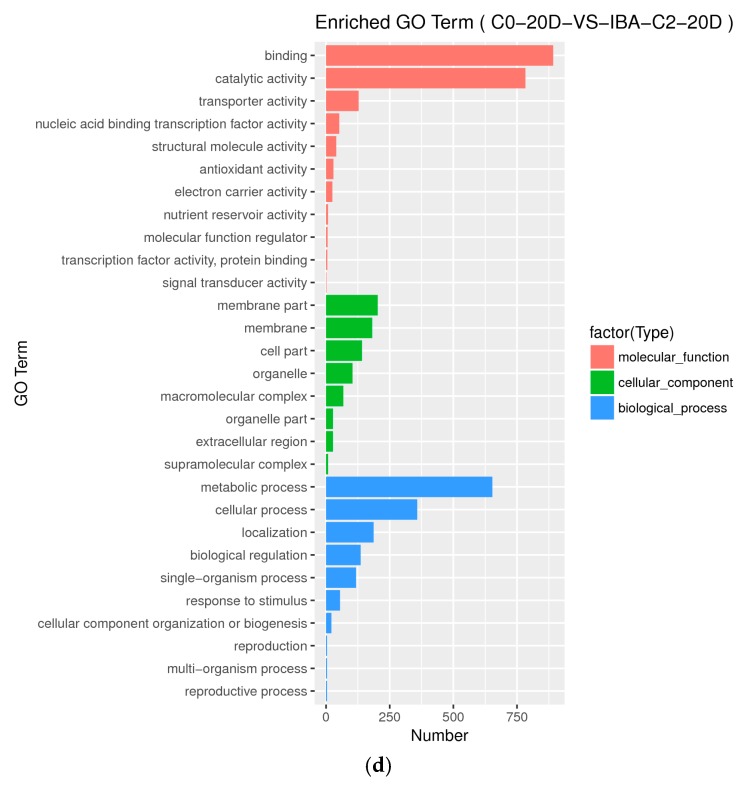
Histogram of Gene Ontology annotation of DEGs from samples treated with different IBA concentrations. (**a**) Enriched GO terms in C0-5D VS IBA-C1-5D; (**b**) Enriched GO terms in C0-5D VS IBA-C2-5D; (**c**) Enriched GO terms in C0-20D VS IBA-C1-20D; (**d**) Enriched GO terms in C0-20D VS IBA-C2-20D. IBA: indole-3-butyric acid; C0: 0 mg dm^−3^ IBA; C1: 0.025 mg dm^−3^ IBA; C2: 0.05 mg dm^−3^ IBA; 0D: 0 day treatment; 5D: 5 days treatment; 20D: 20 days treatment.

GO level secondary gene annotation of differentially expressed genes (DEGs) during somatic embryogenesis in cotton was induced by applying different IBA serial concentration gradients. These results indicate that the relative differential gene expression patterns induced by different IBA concentrations are similar (Figure A2), while the scale of differentially induced genes is greatly different (Table 2).

When comparing different IBA concentrations in the C0-5D VS IBA-C1-5D, C0-5D VS IBA-C2-5D groups (Figure A2a,b) at 5 d when plant hormone IBA was added, the number of differential genes increased with increasing concentrations of IBA. When comparing different IBA concentrations in the C0-20D VS IBA-C1-20D and C0-20D VS IBA-C2-20D groups (Figure A2c,d) at 20 days when plant hormone IBA was added, the number of differential genes decreased with increasing concentrations of IBA. In the same concentration when comparing 5 days with 20 days, the number of differential genes decreased with the increase in culture time.

### 2.8. Differential Metabolic Pathway Analysis

#### 2.8.1. Enriched Metabolic Pathway in Comparison Groups with and without IBA

IBA had a great influence on the differentiation rate, and the callus differentiation rate was very low (7.81%) when IBA was not added. The differentiation rate upon the addition of IBA significantly increased (72.22%) nearly nine-fold.

**Figure 4:**
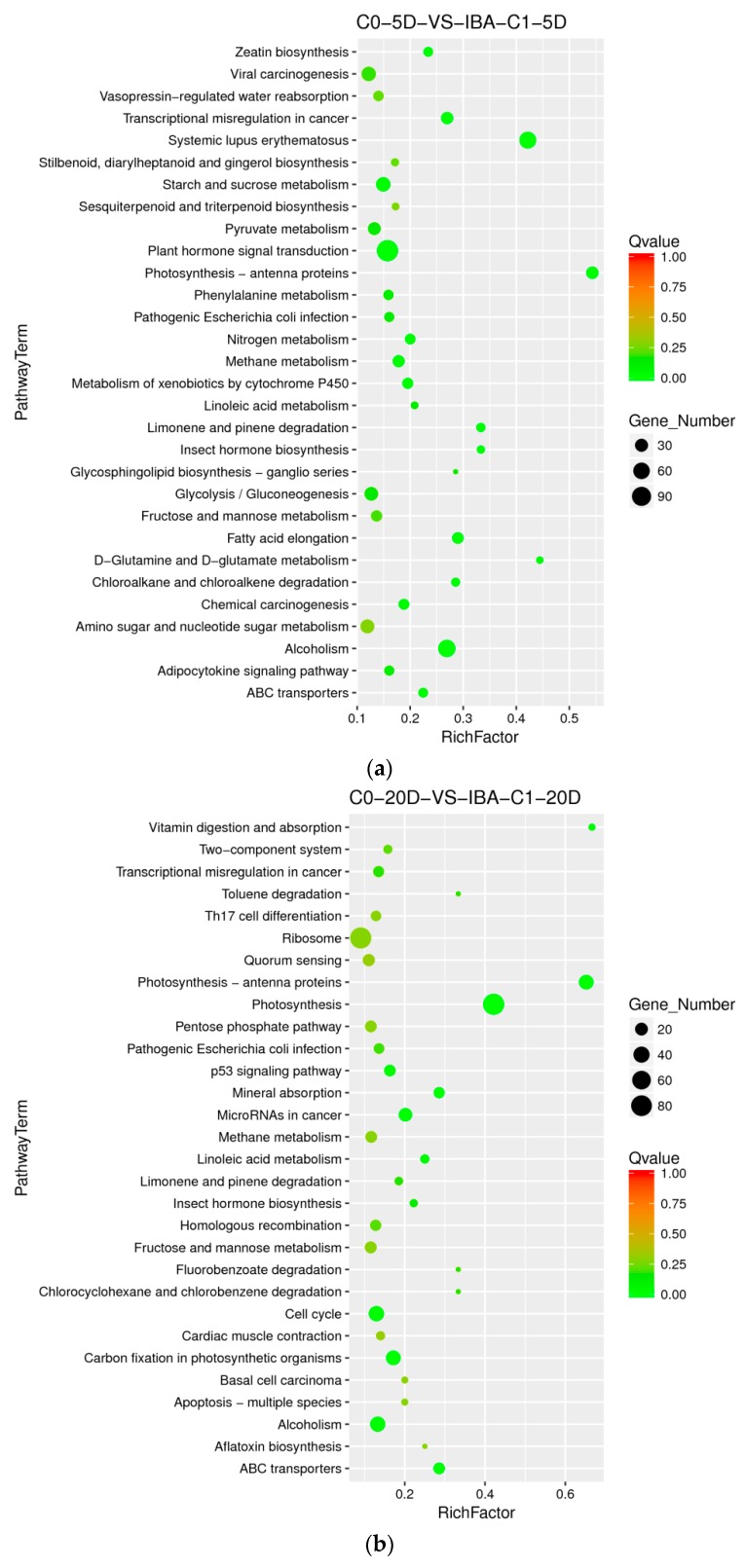
The Kyoto Encyclopedia of Genes and Genomes (KEGG) annotation of DEGs in comparison groups with and without IBA. (**a**) Pathway enrichment in C0-5D VS IBA-C1-5D; (**b**) Pathway enrichment in C0-20D VS IBA-C1-20D. IBA: indole-3-butyric acid; C0: 0 mg dm^−3^ IBA; C1: 0.025 mg dm^−3^ IBA; 5D: 5 days treatment; 20D: 20 days treatment.

To analyze the differential genes related to the IBA-induced cotton callus differentiation rate, we chose a combination of samples treated with IBA and without IBA to find the metabolic pathway that affected the differentiation rate. The high-differentiation combination selected was 0.1 mg dm^−3^ KT+ 0.025 mg dm^−3^ IBA, and the poorly differentiated combination was 0.1 mg dm^−3^ KT + 0 mg dm^−3^ IBA. At 5 d, the metabolic pathways for differentially expressed genes were significantly enriched in plant hormone signal transduction, zeatin biosynthesis, starch and sucrose metabolism, nitrogen metabolism, methane metabolism, metabolism of xenobiotics by cytochrome P450, fatty acid elongation, alcohol degradation, and ABC transporters (Figure 4a). The plant hormone signal transduction pathway was highly enriched at 5 d, indicating that these series endogenous critical corresponding genes were induced and stimulated greatly by external IBA. At 20 days, the pathways with differentially expressed genes were significantly enriched in photosynthetic-antennary proteins, photosynthesis, mineral absorption, cell cycle, carbon fixation in photosynthetic organisms, alcohol degradation, and ABC transporters (Figure 4b). The pathways enriched in photosynthesis, photosynthetic-antennary proteins, and carbon fixation in photosynthetic organisms indicated that genes related to photosynthesis were also essential in the cotton SE, especially at 20 days. The alcohol degradation pathway was significantly enriched in both periods, and these enriched differential genes were upregulated, which suggests that the alcohol degradation pathway may play a positive role in IBA-induced cotton somatic embryogenesis. The differentially expressed genes (photosystem II proteins PSBQ2, PSBS, PSBW, and ferredoxin SEND33) that were significantly enriched in photosynthetic pathways were downregulated at 20 days. Differential genes enriched in cell cycle pathways were upregulated, indicating that the photosynthetic pathway plays a negative regulatory role and the cell cycle pathway plays a positive regulatory role in somatic embryogenesis.

#### 2.8.2. Enriched Differential Metabolic Pathway in Different IBA Doses

**Figure 5:**
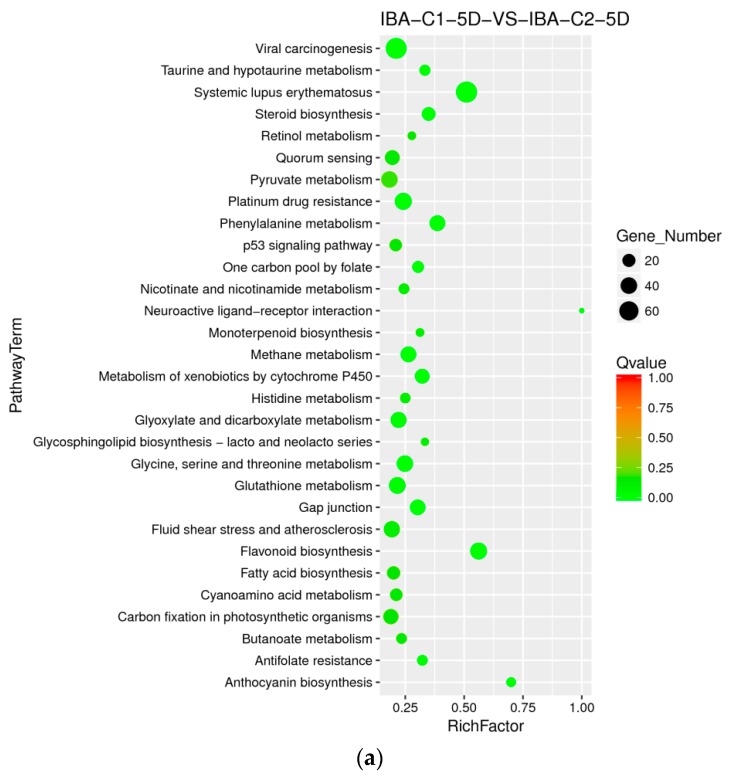

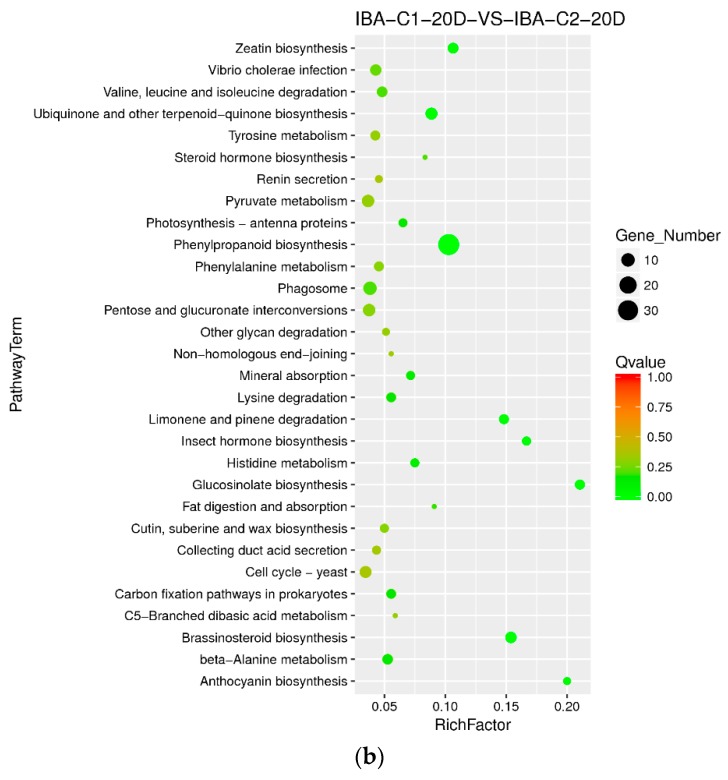
KEGG annotation of differential expressed genes in different IBA doses. (**a**) Pathway enrichment in IBA-C1-5D VS IBA-C2-5D; (**b**) Pathway enrichment in IBA-C1-20D VS IBA-C2-20D. IBA: indole-3-butyric acid; C1: 0.025 mg dm^−3^ IBA; C2: 0.05 mg dm^−3^ IBA; 5D: 5 days treatment; 20D: 20 days treatment.

When adding different concentrations of IBA, the frequency of callus differentiation induced in cotton was also different. Compared with the ideal concentration of 0.025 mg dm^−3^, a high concentration of 0.05 mg dm^−3^ inhibited embryonic differentiation. To analyze the difference in dose effects of IBA, we compared the concentration of 0 mg dm^−3^, 0.025 mg dm^−3^, and 0.05 mg dm^−3^ of IBA and found that the metabolism of xenobiotics by the cytochrome P450 pathway was enriched both in C0-5D VS IBA-C1-5D and IBA-C1-5D VS IBA-C2-5D (Figure 4a, Figure 5a). This indicated that the metabolism of xenobiotics by the cytochrome P450 pathway was dose-dependently expressed. In IBA-C1-5D VS IBA-C2-5D (Figure 5a), the specific enriched pathways were involved in secondary metabolisms such as steroid biosynthesis, flavonoid biosynthesis, and anthocyanin biosynthesis. Corresponding to the phenotypic results, the IBA concentration increases, while the differentiation rate of callus decreases. Therefore, we conclude that the secondary metabolites such as steroids, flavonoids, and anthocyanin may not be conducive to differentiation. At 20 days, photosynthesis-antenna pathway proteins were enriched in both C0-20D VS IBA-C1-20D and IBA-C1-20D VS IBA-C2-20D (Figure 4b, Figure 5b). In IBA-C1-20D VS IBA-C2-20D, the significantly enriched relevant pathways were phenylpropanoid biosynthesis, brassinosteroid biosynthesis and anthocyanin biosynthesis. We speculated that secondary metabolism affects the somatic embryogenesis of cotton. In particular, the results showed that in the IBA-induced cotton calli pre-embryonic initial period (20 days), the phenylpropanoid biosynthesis pathway, which has broad physiological activities and is related to plant growth regulation, was most highly enriched in comparison group of IBA-C1-20D VS IBA-C2-20D.

#### 2.8.3. Enriched Differential Metabolic Pathway with Different Culture Times

**Figure 6:**
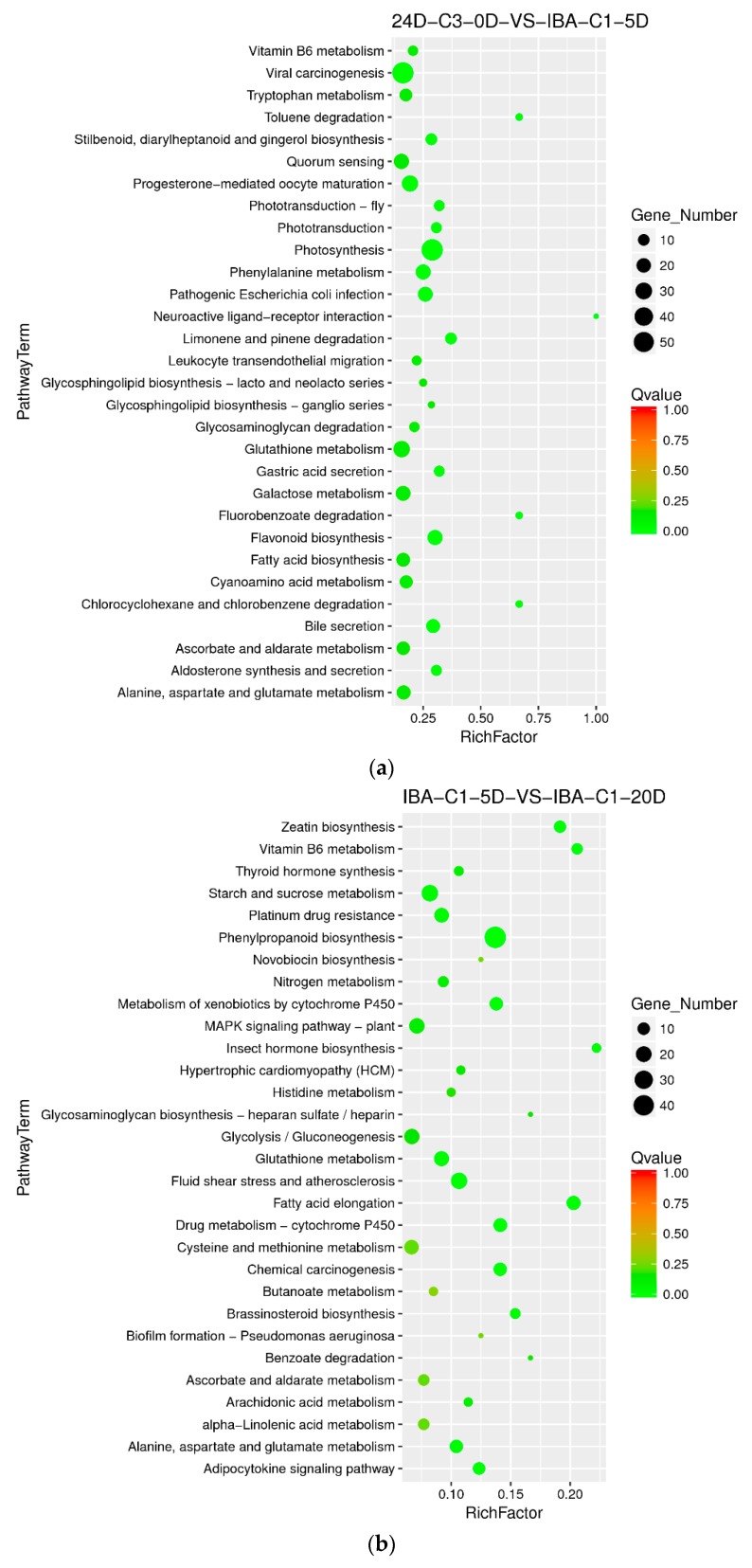

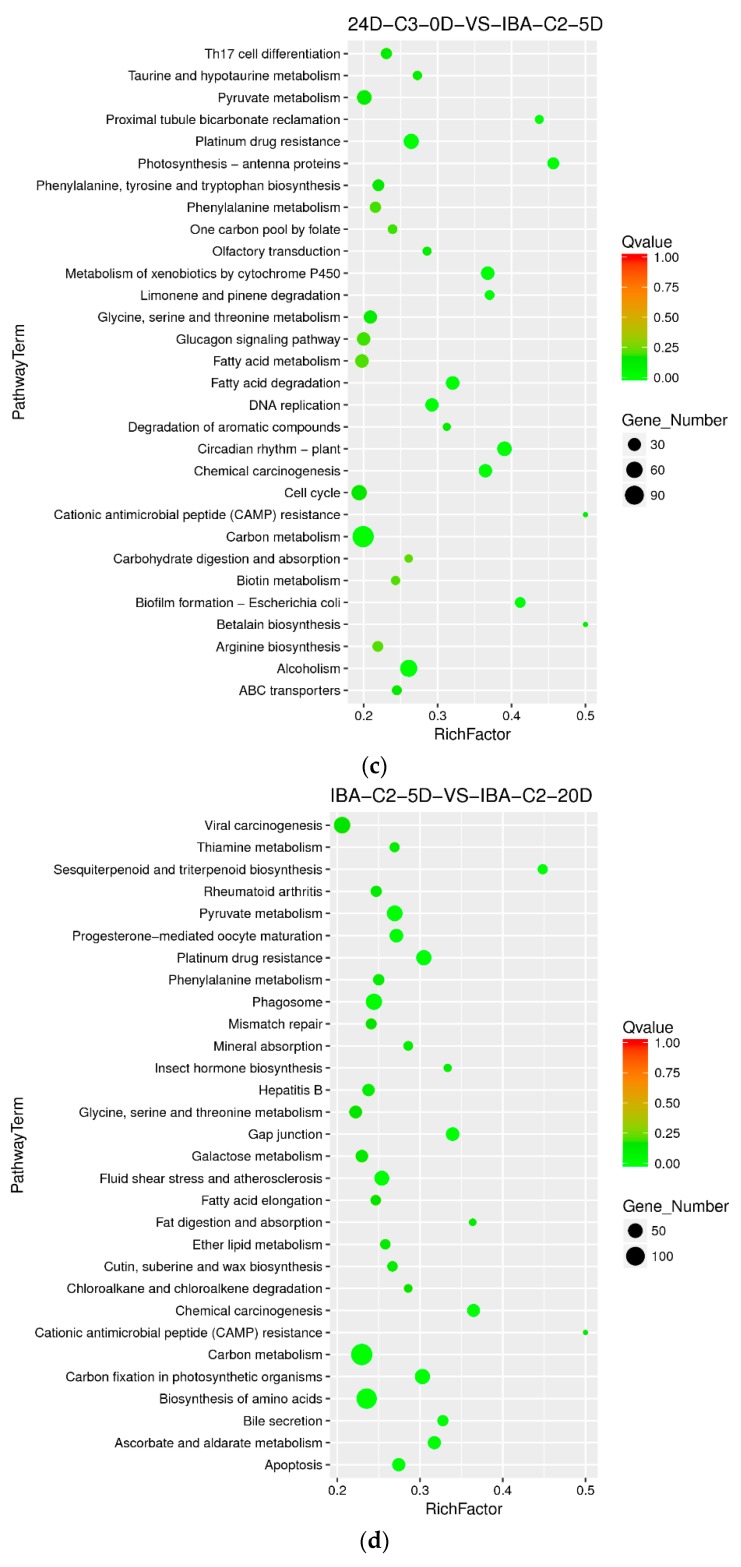
KEGG annotation of differential expressed genes in different culture times of 0 day, 5 days, and 20 days. (**a**) Pathway enrichment in 24D-C3-0D VS IBA-C1-5D; (**b**) Pathway enrichment in IBA-C1-5D VS IBA-C1-20D; (**c**) Pathway enrichment in 24D-C3-0D VS IBA-C2-5D; (**d**) Pathway enrichment in IBA-C2-5D VS IBA-C2-20D. IBA: indole-3-butyric acid; 2, 4-D: 2, 4-dichlorophenoxyacetic acid; C1: 0.025 mg dm^−3^ IBA; C2: 0.05 mg dm^−3^ IBA; C3: 0.1 mg dm^−3^ 2, 4-D; 0D: 0 day treatment; 5D: 5 days treatment; 20D: 20 days treatment.

With the ideal IBA concentration of 0.025 mg dm^−3^, we compared samples at 0 day, 5 days, and 20 days. In the embryogenic responsive stage (5 days), the photosynthesis, flavonoid biosynthesis, phenylalanine metabolism pathways were significantly enriched. And in the pre-embryonic initial period (20 days), the differentially enriched pathways were those of phenylpropanoid biosynthesis, MAPK signaling, glutathione metabolism, fatty acid elongation and brassinosteroid biosynthesis. (Figure 6a,b). Therefore, it is suggested that the pathways above and the corresponding signaling and regulatory genes essentially affect cotton somatic embryogenesis in low IBA concentrations.

When the IBA concentration was 0.05 mg dm^−3^ and we compared samples at 0 day, 5 days and 20 days, it was found that the differentially enriched pathways were those of carbon metabolism, alcohol degradation, fatty acid degradation, circadian rhythm, carbon fixation in photosynthetic organisms, and biosynthesis of amino acids. The results show that in IBA-induced cotton embryogenic redifferentiation (0.05 mg dm^−3^), the carbon metabolism pathway is highly enriched in the comparison groups of 24D-C3-0D VS IBA-C2-5D and IBA-C2-5D VS IBA-C2-20D simultaneously (Figure 6c,d). We speculated that dominant carbon metabolism sequentially affects somatic embryogenesis of cotton at high IBA concentrations.

### 2.9. Expression Profiles of Major Genes Associated with SE Regulation

Transcriptome analysis showed that the complicated and concerted IBA-induced multiple cellular pathways, as well as the corresponding signaling and regulatory genes, are responsible for plant growth regulator-induced SE. We comprehensively explored and mined the significantly representative regulatory pathway and essential genes for cotton SE enriched by KEGG analysis (Table 3), and candidate SE-related crucial transcription factor genes that were not enriched by KEGG (Table 4).

### 2.10. Candidate Gene Expression Pattern Validation

**Figure 7:**
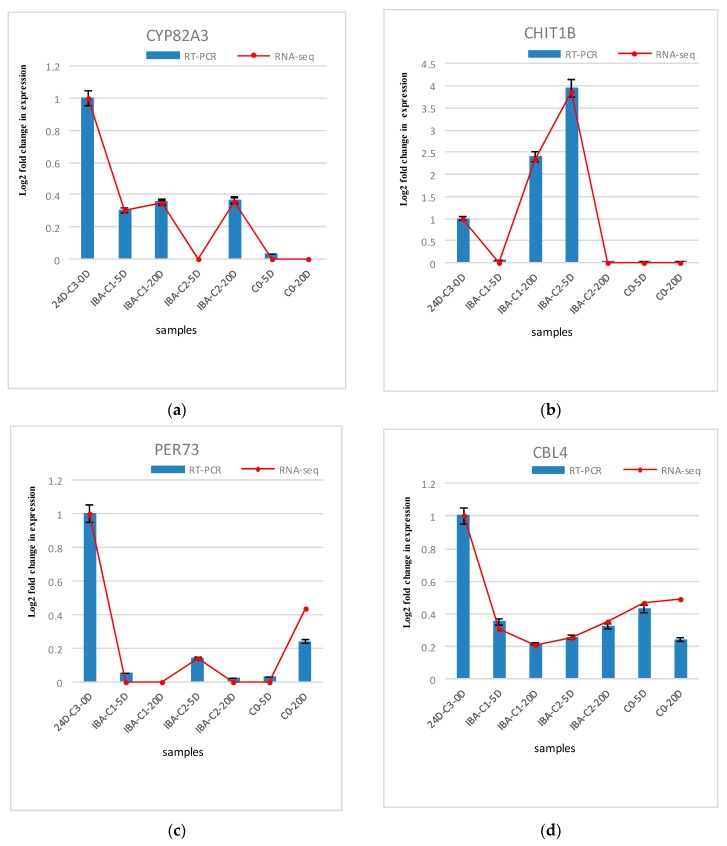

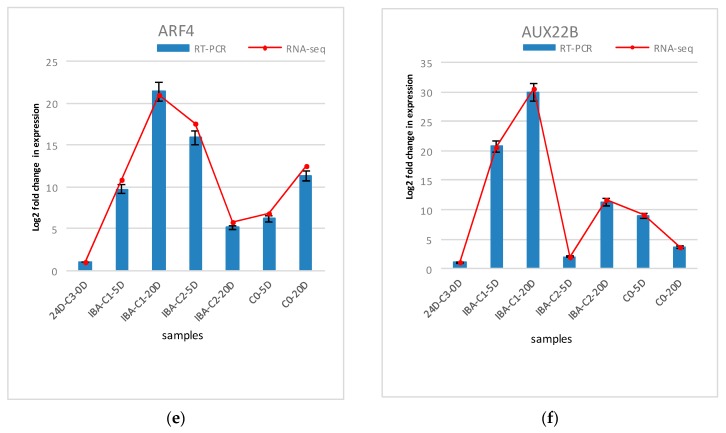
Comparison and confirmation of the RNA-seq data with qRT-PCR. (**a**)–(**f**) Relative expression of DEGs selected by comparative transcriptome induced by IBA. Bars represent SD, and gene abundance was calculated relative to the *GhUB7* expression level.

The differentially expressed genes analyzed in the expression profiling were verified using real-time fluorescence quantitative PCR. Six genes were randomly selected for validation. These six genes include endochitinases (*CHIT1B*), calcineurin B-like protein (*CBL4*), peroxidase (*PER73*), cytochrome P450 (*CYP82A3*), auxin response factor (*ARF4*), and auxin-inducible protein (*AUX22B*). Endochitinase (*CHIT1B*) plays an important role in somatic embryogenesis. For example, carrot embryogenic callus contains glycosylated acidic endochitinase, which can promote somatic embryogenesis. The endochitinase in sugar beet can also promote the early progression of this process. It is suggested that endochitinase is related to some somatic embryogenesis signal molecules. Calcineurin B-like protein (*CBL4*) was selected from the calcium signaling pathway. *CBLs* represent a family of plant calcium-binding proteins that function in calcium signaling by interacting with their interacting protein kinases. These roles are essential for plant growth and development. Cytochrome P450 (*CYP82A3*) is enriched in the brassinosteroid biosynthesis pathway. Peroxidase (*PER73*) was selected from phenylpropanoid biosynthesis pathway. Peroxidase is considered to take part in diverse plant processes, such as auxin metabolism, cell wall elongation and stiffening, and it is also related to different metabolic pathways to regulate somatic embryogenesis [29,30]. *ARF4* and *AUX22B* genes were selected from the plant hormone signal transduction pathway, which are the two key transcription factors involved in regulating the expression of auxin-responsive genes. Auxin response factor (*ARFs*) is considered as essential SE genes that are specifically activated during embryogenic differentiation [31,32,33,34]. ARFs bind auxin response promoter elements, mediate transcriptional responses to auxin and regulate auxin-mediated transcriptional activation/repression, together with *Aux/IAA* [35,36]. Three repetitions of the biological experiments were performed. The results are shown in Figure 7. The results of the fluorescent quantitative PCR analysis are basically consistent with the gene expression patterns obtained by RNA-sequencing, indicating that the transcriptome data are reliable and that these genes are regulated during hormone-induced somatic embryogenesis in cotton. Their role can be determined by further studying their function.

## 3. Discussion

Somatic embryogenesis (SE) is a very complex biological process that is affected by genotypes, hormones, stress, light, mineral elements, nitrogen sources, and carbon sources in the medium. However, somatic cells under the influence of various internal and external factors initiate the expression of certain specific genes and then redifferentiate into embryonic cells. Comparative transcriptome analysis is useful to dissect the molecular mechanism of SE. The differentiation ability of plants is affected by the interaction of two factors in vivo and in vitro. Therefore, this experiment detected endogenous gene changes through exogenous IBA treatment and tried to find endogenous factors responding to exogenous IBA changes, to provide a reliable theoretical and experimental basis for cotton somatic embryogenesis.

### 3.1. IBA Embryogenic Inductive Effect and Dose Effect During Differentiation of Cotton SE

In this experiment, we found that different concentrations of IBA had different effects on embryonic differentiation, as demonstrated by the differentiation rate statistics in cotton calli. The results (Table 1) show that the combination of 0.1 mg dm^−3^ KT + 0.025 mg dm^−3^ IBA induced the highest differentiation rate of cotton. Our callus differentiation rate statistics for exogenously applied auxin and cytokinin were consistent with previous studies, i.e., the induction of EC and SEs, and the concentration of 2, 4-D in the medium was replaced by a combination of IBA and KT [24,37]. In this experiment, the role of auxin and cytokinin was not achieved alone, but by interacting with other pathways and components involved in the differentiation and development of SEs, such as their upstream TF regulators and hormone signaling pathways [38]. Wang et al. [39] found that, in cotton tissue cultures, the effect of IBA is stronger than that of the other auxins or auxin analogues. Comparing 0.025 mg dm^−3^ and 0.05 mg dm^−3^, the high concentration of IBA inhibited callus differentiation. Under 0.1 mg dm^−3^ KT medium, the differentiation rate was lowest, further indicating that cytokinin alone inhibited cell differentiation and that appropriate ratio between auxin and cytokinin is necessary in callus culture. Moreover, these results indicate that the appropriate concentration of IBA is conducive to callus differentiation.

The investigation in this study shows that a series of different IBA concentrations induced similar relative differential gene expression patterns (Figure A2), while the concentrations had different induction rates in cotton somatic embryogenesis (Table 1) and the amount of differentially induced genes were greatly different (Table 2). To resolve different IBA concentration induction rates, we analyzed the different IBA comparison groups under 0 mg dm^−3^, 0.025 mg dm^−3^, and 0.05 mg dm^−3^ concentrations. We found that the metabolism of xenobiotics by the cytochrome P450 and photosynthetic pathway shows a dose-dependent pattern. Meanwhile, the secondary metabolism and phenylpropanoid biosynthesis pathways were significantly enriched. These results indicate that these pathways could essentially affect the somatic embryogenesis of cotton. Previous studies have shown that secondary metabolites in the embryogenic callus are lower than in the nonembryonic callus. This indicates that secondary metabolism is relatively vigorous in a nonembryonic callus, which may affect the main metabolic rate and intensity and, ultimately, the cell differentiation, which is consistent with our findings. Especially, highly enriched phenylpropanoid biosynthesis in our study may play an important role in the SE. The data were consistent with and extended the broad physiological activities and related to plant growth regulation of phenylpropanoid metabolism.

### 3.2. Plant Hormone Signal Transduction and Alcohol Degradation Pathway in Embryogenic Induction Responsive Stage

The exogenous hormone is essential during cotton somatic embryogenesis [40]. The results in this study show that IBA has a great influence on the differentiation rate, and that the plant hormone signal transduction pathway is significantly enriched in the embryogenic induction responsive stage. It is suggested that the plant growth regulator IBA response generally involves initiating signals through receptor binding, transducing signals through complex downstream molecular events, and inducing changes in cell morphology and metabolism to achieve signal output. The signal of exogenous IBA is recognized by a receptor, thus triggering intracellular transduction and amplification of the signal, and will ultimately lead to cellular and organ responses. Hormone production, signaling and responses can affect plant growth and development, which are of high importance for cotton somatic embryogenesis.

In 1991, Perata P and Alpi A [41] detected the formation of 14C carbon dioxide by adding 14C-labeled ethanol to carrot cell suspensions. They thus deduced that carrot cells had the ability to degrade ethanol. Later, the scientists found that the tobacco callus is able to digest and metabolize ethanol. At the time of cultivation, the tobacco callus converts ethanol into acetic acid, which is almost harmless to plants [42]. Further experiments have also found that plant hormones have a significant regulatory effect on ethanol digestion and metabolism of plants. In this study, when the high differentiation rate IBA combination (0.025 mg dm^−3^ IBA) was compared with the low differentiation rate IBA combination (0 mg dm^−3^ IBA), we found that the alcohol degradation pathway was significantly enriched in both 5 days and 20 days cultures (Figure 4), and the genes enriched in this pathway were upregulated. We speculated that the alcohol degradation pathway plays a positive role in the regulation of IBA-induced cotton callus differentiation, which is consistent with previous studies.

### 3.3. Photosynthesis, Cell Cycle and SERK Pathway During Somatic Embryogenesis in Cotton

When the high differentiation rate of IBA combination (0.025 mg dm^−3^ IBA) was compared with the low differentiation rate of IBA combination (0 mg dm^−3^ IBA), the differentially expressed genes (the photosystem II proteins *PSBQ2*, *PSBS*, *PSBW* and ferredoxin *SEND33*) that were significantly enriched in the photosynthesis pathway were downregulated at 20 days of culture. The differentially expressed genes enriched in the cell cycle pathway (cell division regulatory protein sna41, *CDC6B*, DNA replication licenser *MCM3*, cell division cycle protein *CDC20-1*) were upregulated, indicating that photosynthesis may play a negative regulatory role in somatic embryogenesis, and that the cell cycle plays a positive regulatory role.

Some of the identified genes have been experimentally demonstrated to play an important role in SE [29,35,43,44]. Somatic embryogenesis receptor kinase (*SERK*) is involved in the signal transduction pathway during somatic embryogenesis and is a marker gene for the embryotic state. Schmidt et al. [45] screened a key gene regulating the transformation of somatic cells into embryonic cells in the hypocotyl regeneration of carrots, and this gene is named *SERK1* (somatic embryogenesis receptor protein kinase 1). In this study, *GhSERK1* (Gh_A01G0158) was found in four comparison groups, including 24D-C3-0D VS C0-5D, 24D-C3-0D VS IBA-C1-5D, 24D-C3-0D VS IBA-C1-20D, and 24D–C3-0D VS IBA-C2-20D. It was up-regulated in all of them, indicating that the up-regulated expression of SERK plays an important role in cotton somatic embryogenesis induction.

## 4. Materials and Methods

### 4.1. Plant Materials and Culture Conditions

One cotton accession, the tetraploid upland cotton species cultivar YZ-1 (Institute of Cotton Research of the Chinese Academy of Agricultural Sciences (CAAS)), which has medium to high somatic embryogenesis, was used for the study. Seeds of the cotton cultivar YZ-1, after uncovering the coats, were sterilized in 0.1% (*w*/*v*) HgCl_2_ (Beijing chemical Co. Ltd, Beijing China) solution for 10 min, then rinsed four times with sterilized distilled water and germinated on a 1/2 MS (Murashige and Skoog) (Qingdao Hope Bio-Technology Co. Ltd, Qingdao, China) medium containing 1.5% (*w*/*v*) sucrose (Hushi Co. Ltd, Shanghai, China) and 0.25% (*w*/*v*) phytagel (Sigma, St. Louis, MO, USA). Callus initiation was conducted with 5 to 7 mm long hypocotyl sections of YZ-1, as described by Wu et al. [12], in the dedifferentiation induction medium containing MS+ 0.1 mg dm^−3^ KT (Sigma-Aldrich, Co., St. Louis, MO, USA, purity ≥ 99.0%) + 0.1 mg dm^−3^ 2, 4-D (Sigma-Aldrich, Co., St. Louis, MO, USA, purity ≥ 97.0%) + 30 g dm^−3^ sucrose+ 2.5 g dm^−3^ phytagel [46]. Additionally, among the 24D-C3-0D, C3 remarked the concentration of 0.1 mg dm^−3^ KT+ 0.1 mg dm^−3^ 2, 4-D. Following callus initiation and six weeks of callus proliferation, the calli were subsequently isolated and subcultured in the redifferentiation medium with 0.1 mg dm^−3^ KT and different degraded exogenous IBA (Sigma-Aldrich, Co., St. Louis, MO, USA, purity ≥ 99.0%) concentrations to induce somatic cell development program reconstruction and embryonic redifferentiation, as described by Zeng et al. [4]. For IBA concentrations, a series of descending concentration gradients were used (0 mg dm^−3^, 0.025 mg dm^−3^, and 0.05 mg dm^−3^) during embryogenic callus induction process. The pH of the medium was 6.0. Primary dedifferentiated calli were respectively sampled at 0 day, 5 days and 20 days after callus initiation. All experimental treatments were set up for three biological replications.

### 4.2. RNA Extraction, cDNA Library Preparation, and RNA-Seq

The total RNA of each sample was extracted by using the EASYspin Plus plant RNA rapid extraction kit (Aidlab Biotechnologies Co. Ltd., Beijing, China), following the manufacturer’s protocol. The total RNA of each sample was quantified and verified with an Agilent 2100 Bioanalyzer (Agilent Technologies, Santa Clara, CA, USA), NanoDrop2000 (Thermo Fisher Scientific, Inc, Waltham, MA, USA) and 1% agarose gel electrophoresis. Next-generation sequencing libraries were prepared using NEBNext ^®^ Ultra™ RNA Library Prep Kit for Illumina^®^ (NEB, USA), following the manufacturer’s instructions. After library purification (Beckman Agencourt AMPure XP beads), the PCR products were cleaned up using AxyPrep Mag PCR Clean-up (Axygen, Union City, CA), validated using an Agilent 2100 Bioanalyzer, and quantified by Qubit 2.0 Fluorometer (Invitrogen, Carlsbad, CA, USA). Then, the libraries with different indices were multiplexed and loaded on an Illumina HiSeq instrument according to the manufacturer’s instructions (Illumina, San Diego, CA, USA). Sequencing was carried out using a 2 × 150 bp paired-end configuration; image analysis and base calling were conducted using HiSeq Control Software (v2.0.5, HCS, Illumina, San Diego, CA, USA), Off-Line Base caller and GAPipeline-1.6 on the HiSeq instrument.

### 4.3. Mapping and Differential Expression Analysis

The filtered sequencing clean data were compared with the upland cotton reference genome (https://www.cottongen.org/species/Gossypium_hirsutum/nbi-AD1_genome_v1.1). Hisat2 (v2.0.1) was used to index the reference genome sequences. First, transcripts in fasta format were converted from known gff annotation files and indexed properly. Then, with the file as a reference gene file, HTSeq (v0.6.1) estimated the gene and isoform expression levels from the pair-end clean data. RPKM (Reads Per Kilo bases per Million reads) was applied to calculate the level of the gene expression indifferent groups. The read counts were normalized with DESeq2 (v1.6.3, open source, http://www.bioconductor.org/) [47]. Differentially expressed genes (DEGs) were defined as those with ≥2-fold change and FDR ≤ 0.05. The normalized sequencing data of three replicates with repeatability were used for analysis.

### 4.4. GO Functional Annotation and KEGG Enrichment Analysis

Gene Ontology (GO) terms describe cellular components, biological processes, and molecular functions of gene sets from differentially expressed genes. GO-TermFinder (v0.86, http://search.cpan.org/dist/GO-TermFinder/) was used to identify GO terms that annotate a list of enriched genes with a significant P-value of less than 0.05. KEGG (Kyoto Encyclopedia of Genes and Genomes) is a collection of databases dealing with genomes, biological pathways, diseases, drugs, and chemical substances (http://en.wikipedia.org/wiki/KEGG). We used scripts in house to determine which KEGG pathways the significantly differentially expressed genes were in.

### 4.5. Alternative Splicing Analysis

ASprofile v1.0.4 is a suite of programs for extracting, quantifying and comparing alternative splicing (AS) events from RNA-seq data. It took a GTF transcript file created by Cufflinks [48] as its input.

### 4.6. Validation of RNA-Seq Data by Real-Time RT-PCR

To verify the differentially expressed genes detected by the Illumina RNA-Seq data, real-time RT-PCR (qRT-PCR) was performed on a set of 3 replicates for each sample and the expression levels calculated were based on three technical replicates. Six genes were chosen for digital gene expression analysis. The gene-specific primers (Table A1) were designed using Primer Premier 5.0 software (http://www.premierbiosoft.com/primerdesign/, Premier Biosoft International, Palo Alto, CA, USA) and synthesized by the Shanghai Shenggong Company. The length of the products ranged was 100–300 bp. The cDNA was synthesized using EasyScript One-Step gDNA Removal and cDNA Synthesis SuperMix (TransGen Biotech, Beijing, China) in a 20 mm^3.^ reaction mixture according to the manufacturer’s instructions. For reverse transcription, an RNA-amount of 5 μg was used. A total of 20 mm^3^ reaction system as follows: Total RNA/mRNA 7 mm^3^, Anchored Oligo (dT)_18_ Primer (0.5 μg mm^−3^) 1 mm^3^, 2 × TS Reaction mix 10 mm^3^, Tran Script RT/RI Enzyme Mix 1 mm^3^, gDNA Remover 1 mm^3^. The first step was to remove gDNA: added 5 × gDNA Buffer, Total RNA, Rnase-Free ddH_2_O, 65 °C incubation 5 min. Then, the solution with Anchored Oligo(dT)_18_ Primer, 2 × TS Reaction mix, and Tran Script RT/RI Enzyme Mix added, was incubated at 42 °C for 15 min. Following this step, Trans Script RT/RI and gDNA Remover enzyme were heat-inactivated at 85 °C for 5 s and subsequently chilled on ice. The obtained cDNA was either subsequently processed or stored at −20 °C until use. qRT-PCR was performed in 15 mm^3^ reactions on the Real-Time PCR Thermal Cycler (Analytik Jena AG, qTOWER^3^ G, Germany), using 1.5 mm^3^ of first-strand cDNA as the template, 7.5 mm^3^ of 2 × UltraSYBR Mixture (with ROX) (CWBIO, Beijing, China), 1.2 mm^3^ each of 10 μM forward and reverse gene-specific primer and 3.6 mm^3^ of ddH2O. *GhUB7* was used as the reference gene. The qRT-PCR conditions were as follows; pre-incubation at 95 °C for 10 min, followed by amplification by 38 cycles at 95 °C for 15 s, 60 °C for 30 s, and 72 °C for 30 s. A melting curve analysis was conducted to evaluate the primer specificity for each primer set to verify the presence of a single melting peak after amplification. The melting curve analysis conditions were as follows: 95 °C 15 s, 60 °C 1 min, 95 °C 15 s, 60 °C 15 s.

## Figures and Tables

**Table 1:** Statistics of callus differentiation rate of cotton YZ-1.

IBA Concentration (mg dm^−3^)	KT Concentration (mg dm^−3^)	Callus Differentiation Rate (%)
0.05 (C2)	0.1	52.63
0.025 (C1)	0.1	72.22
0 (C0)	0.1	7.81

**Table 2:** Summary of annotated genes in each database of the pairwise comparisons.

Pairwise Comparisons	NR	GO	KEGG
24D-C3-0D VS IBA-C1-5D	7264	5007	1789
24D-C3-0D VS IBA-C1-20D	5141	3507	1300
24D-C3-0D VS IBA-C2-5D	9486	6444	2524
24D-C3-0D VS IBA-C2-20D	4314	2930	1080
24D-C3-0D VS C0-5D	2135	1434	550
24D-C3-0D VS C0-20D	5349	3731	1415
IBA-C1-5D VS IBA-C1-20D	2924	2036	723
IBA-C2-5D VS IBA-C2-20D	11,235	7564	2828
C0-5D VS C0-20D	4118	2785	1191
IBA-C1-5D VS IBA-C2-5D	8889	6068	2318
IBA-C1-20D VS IBA-C2-20D	1542	1038	333
C0-5D VS IBA-C1-5D	6181	4269	1518
C0-5D VS IBA-C2-5D	10,227	6926	2685
C0-20D VS IBA-C1-20D	4404	2994	1244
C0-20D VS IBA-C2-20D	2363	1626	668

**Table 3:** Significantly representative differentially expressed genes were enriched by KEGG.

No.	Gene ID	Gene Description	Pathway Annotation	*p*-Value	Regulation	Compare Combination
1	Gh_A05G0085	EIN3-binding F-box protein 1-like isoform X2	plant hormone signal transduction	4.06 × 10^−84^	Down	C0-5D VS IBA-C1-5D
2	Gh_D05G0148	EIN3-binding F-box protein 1-like isoform X2	plant hormone signal transduction	1.08 × 10^−67^	Down	C0-5D VS IBA-C1-5D
3	Gh_D09G0971	pathogenesis-related protein 1-like	plant hormone signal transduction	1.87 × 10^−55^	Down	C0-5D VS IBA-C1-5D
4	Gh_D06G1764	abscisic acid receptor PYL4-like	plant hormone signal transduction	4.05 × 10^−55^	Down	C0-5D VS IBA-C1-5D
5	Gh_A06G1418	abscisic acid receptor PYL4-like	plant hormone signal transduction	6.63 × 10^−41^	Down	C0-5D VS IBA-C1-5D
6	Gh_D12G0182	probable indole-3-acetic acid-amido synthetase GH3.1	plant hormone signal transduction	1.15 × 10^−38^	Ups	C0-5D VS IBA-C1-5D
7	Gh_D08G0034	histone H2A-like	alcohol degradation	4.07 × 10^−24^	Ups	C0-5D VS IBA-C1-5D
8	Gh_D09G0854	histone H2B-like	alcohol degradation	4.91 × 10^−18^	Ups	C0-5D VS IBA-C1-5D
9	Gh_D02G1958	histone deacetylase 19 isoform X1	alcohol degradation	5.05 × 10^−16^	Ups	C0-5D VS IBA-C1-5D
10	Gh_A09G1018	G2/mitotic-specific cyclin S13-7	cell cycle	1.47 × 10^−15^	Ups	C0-20D VS IBA-C1-20D
11	Gh_D09G1039	G2/mitotic-specific cyclin S13-7	cell cycle	2.00 × 10^−6^	Ups	C0-20D VS IBA-C1-20D
12	Gh_D11G0102	cell division cycle 20.2, cofactor of APC complex-like	cell cycle	2.08 × 10^−6^	Ups	C0-20D VS IBA-C1-20D
13	Gh_A05G2161	cyclin-B2-4-like isoform X2	cell cycle	3.36 × 10^−6^	Ups	C0-20D VS IBA-C1-20D
14	Gh_A08G1487	probable serine/threonine-protein kinase cdc7 isoform X1	cell cycle	5.93 × 10^−6^	Ups	C0-20D VS IBA-C1-20D
15	Gh_D08G0847	putative cyclin-B3-1	cell cycle	1.65 × 10^−5^	Ups	C0-20D VS IBA-C1-20D
16	Gh_A08G2544	histone H2A	alcohol degradation	1.48 × 10^−23^	Ups	C0-20D VS IBA-C1-20D
17	Gh_A09G0836	probable histone H2B.1	alcohol degradation	6.06 × 10^−8^	Ups	C0-20D VS IBA-C1-20D
18	Gh_D05G0853	histone H2B-like	alcohol degradation	4.01 × 10^−7^	Ups	C0-20D VS IBA-C1-20D
19	Gh_A11G1738	oxygen-evolving enhancer protein 1, chloroplastic	photosynthesis	4.66 × 10^−54^	Down	C0-20D VS IBA-C1-20D
20	Gh_D11G1768	ferredoxin--NADP reductase, leaf isozyme, chloroplastic	photosynthesis	4.87 × 10^−54^	Down	C0-20D VS IBA-C1-20D
21	Gh_D07G1624	photosystem I reaction center subunit XI, chloroplastic-like	photosynthesis	6.18 × 10^−54^	Down	C0-20D VS IBA-C1-20D
22	Gh_D05G1742	plastocyanin	photosynthesis	6.16 × 10^−50^	Down	C0-20D VS IBA-C1-20D
23	Gh_A02G1466	peroxidase 73-like	Phenylpropanoid biosynthesis	1.25 × 10^−283^	Down	IBA-C1-5D VS IBA-C2-5D
24	Gh_A09G1415	peroxidase 21	Phenylpropanoid biosynthesis	4.58 × 10^−248^	Down	IBA-C1-5D VS IBA-C2-5D
25	Gh_A08G0747	peroxidase 16-like	Phenylpropanoid biosynthesis	3.63 × 10^−226^	Down	IBA-C1-5D VS IBA-C2-5D
26	Gh_D08G0829	peroxidase 59	Phenylpropanoid biosynthesis	4.52 × 10^−207^	Ups	IBA-C1-5D VS IBA-C2-5D
27	Gh_D02G2386	cytochrome P450 CYP749A22-like isoform X1	Brassinosteroid biosynthesis	5.39 × 10^−19^	Down	IBA-C1-5D VS IBA-C1-20D
28	Gh_D08G2495	cytochrome P450 734A1-like	Brassinosteroid biosynthesis	3.35 × 10^−17^	Ups	IBA-C1-5D VS IBA-C1-20D
29	Gh_A06G1446	cytochrome P450 90A1	Brassinosteroid biosynthesis	7.63 × 10^−5^	Down	IBA-C1-5D VS IBA-C1-20D
30	Gh_D02G0227	crocetin glucosyltransferase, chloroplastic-like	Anthocyanin biosynthesis	2.70 × 10^−63^	Down	IBA-C1-5D VS IBA-C1-20D
31	Gh_D02G0225	crocetin glucosyltransferase, chloroplastic-like	Anthocyanin biosynthesis	1.09 × 10^−60^	Down	IBA-C1-5D VS IBA-C1-20D
32	Gh_D02G0230	crocetin glucosyltransferase, chloroplastic-like	Anthocyanin biosynthesis	1.33 × 10^−28^	Down	IBA-C1-5D VS IBA-C1-20D
33	Gh_A09G1509	glutathione S-transferase U8-like	Metabolism of xenobiotics by cytochrome P450	2.76 × 10^−118^	Ups	IBA-C1-5D VS IBA-C2-5D
34	Gh_D01G0907	alcohol dehydrogenase-like 7	Metabolism of xenobiotics by cytochrome P450	2.01 × 10^−44^	Down	IBA-C1-5D VS IBA-C2-5D
35	Gh_D02G2184	squalene synthase-like	Steroid biosynthesis	1.43 × 10^−63^	Down	IBA-C1-5D VS IBA-C2-5D
36	Gh_D04G2024	squalene monooxygenase-like	Steroid biosynthesis	9.41 × 10^−58^	Ups	IBA-C1-5D VS IBA-C2-5D
37	Gh_D06G0846	3beta-hydroxysteroid-dehydrogenase/decarboxylase isoform 2 isoform X1	Steroid biosynthesis	1.22 × 10^−23^	Down	IBA-C1-5D VS IBA-C2-5D
38	Gh_A05G1647	dihydroflavonol-4-reductase-like	Flavonoid biosynthesis	2.65 × 10^−37^	Down	IBA-C1-5D VS IBA-C2-5D
39	Gh_D12G1798	Flavonoid 3’-monooxygenase	Flavonoid biosynthesis	3.84 × 10^−34^	Down	IBA-C1-5D VS IBA-C2-5D
40	Gh_D03G0311	phosphoenolpyruvate carboxykinase [ATP]	Carbon metabolism	8.18 × 10^−125^	Down	24D-C3-0D VS IBA-C2-5D
41	Gh_D07G0203	ATP-dependent 6-phosphofructokinase 6 isoform X1	Carbon metabolism	3.85 × 10^−90^	Ups	24D-C3-0D VS IBA-C2-5D
42	Gh_D05G3328	malate dehydrogenase, mitochondrial	Carbon metabolism	2.09 × 10^−57^	Down	24D-C3-0D VS IBA-C2-5D
43	Gh_D02G1553	glyceraldehyde-3-phosphate dehydrogenase, cytosolic	Carbon metabolism	1.02 × 10^−259^	Ups	IBA-C2-5D VS IBA-C2-20D
44	Gh_A02G0386	malate dehydrogenase	Carbon metabolism	1.12 × 10^−177^	Ups	IBA-C2-5D VS IBA-C2-20D

**Table 4:** Differentially expressed transcription factor genes were not enriched by KEGG in cotton SE.

No.	Gene ID	Gene Description	*p*-Value	Regulation	Compare Combination
1	Gh_D05G1962	WUSCHEL-related homeobox 4-like	1.37 × 10^−12^	Ups	C0-20D-VS-IBA-C1-20D
2	Gh_D11G3261	WUSCHEL-related homeobox 8-like	4.35 × 10^−3^	Ups	C0-5D-VS-IBA-C1-5D
3	Gh_D05G1503	WUSCHEL-related homeobox 9-like	1.76 × 10^−31^	Down	C0-5D-VS-IBA-C1-5D
4	Gh_D10G0270	WUSCHEL-related homeobox 9-like isoform X1	2.31 × 10^−6^	Down	C0-5D-VS-IBA-C1-5D
5	Gh_D05G1962	WUSCHEL-related homeobox 4-like	1.37 × 10^−12^	Ups	C0-5D-VS-IBA-C1-5D
6	Gh_A07G1368	zinc finger protein ZAT9-like	4.07 × 10^−6^	Down	C0-20D-VS-IBA-C1-20D
7	Gh_A09G0899	zinc finger protein WIP3-like	1.76 × 10^−5^	Ups	C0-20D-VS-IBA-C1-20D
8	Gh_D11G1518	zinc finger protein CONSTANS-LIKE 9-like isoform X1	6.71 × 10^−6^	Ups	C0-20D-VS-IBA-C1-20D
9	Gh_D12G0712	zinc finger protein AZF1-like	2.55 × 10^−5^	Ups	C0-5D-VS-IBA-C1-5D
10	Gh_A01G0135	zinc finger CCCH domain-containing protein 2-like	3.94 × 10^−6^	Down	C0-5D-VS-IBA-C1-5D/C0-20D-VS-IBA-C1-20D
11	Gh_D01G0181	zinc finger CCCH domain-containing protein 2-like	1.17 × 10^−5^	Down	C0-20D-VS-IBA-C1-20D
12	Gh_D11G0511	transcription factor MYB44-like	2.63 × 10^−47^	Down	C0-5D-VS-IBA-C1-5D
13	Gh_A06G0188	transcription factor MYB44-like	2.21 × 10^−66^	Down	C0-5D-VS-IBA-C1-5D/ C0-20D-VS-IBA-C1-20D
14	Gh_A09G0939	transcription factor LHW	1.88 × 10^−47^	Ups	C0-5D-VS-IBA-C1-5D/ C0-20D-VS-IBA-C1-20D
15	Gh_D02G0841	transcription factor bHLH96-like	9.80 × 10^−56^	Down	C0-5D-VS-IBA-C1-5D
16	Gh_D07G0897	transcription factor bHLH62-like	2.12 × 10^−69^	Down	C0-20D-VS-IBA-C1-20D
17	Gh_A07G2268	transcription factor bHLH62-like	3.76 × 10^−46^	Down	C0-5D-VS-IBA-C1-5D
18	Gh_D07G0897	transcription factor bHLH62-like	2.12 × 10^−69^	Down	C0-5D-VS-IBA-C1-5D
19	Gh_D11G3340	transcription factor bHLH106	4.72 × 10^−71^	Ups	C0-5D-VS-IBA-C1-5D
20	Gh_A09G0433	somatic embryogenesis receptor kinase 5	3.30 × 10^−4^	Ups	IBA-C1-5D-VS-IBA-C2-5D
21	Gh_D09G0447	somatic embryogenesis receptor kinase 5	1.28 × 10^−4^	Ups	C0-20D-VS-IBA-C1-20D
22	Gh_A01G0158	somatic embryogenesis receptor kinase 2-like isoform X1	3.31 × 10^−4^	Ups	C0-20D-VS-IBA-C1-20D
23	Gh_D07G1063	serine/threonine-protein kinase D6PKL2-like	1.04 × 10^−88^	Down	C0-5D-VS-IBA-C1-5D
24	Gh_A10G1074	receptor-like protein kinase 2	1.77 × 10^−54^	Down	C0-5D-VS-IBA-C1-5D
25	Gh_D10G1439	receptor-like protein kinase 2	4.76 × 10^−86^	Down	C0-20D-VS-IBA-C1-20D
26	Gh_A09G1432	putative receptor protein kinase ZmPK1 isoform X1	1.58 × 10^−53^	Ups	C0-5D-VS-IBA-C1-5D
27					
28	Gh_A06G0601	probable nuclear hormone receptor HR38 isoform X2	3.92 × 10^−3^	Down	C0-5D-VS-IBA-C1-5D
29	Gh_D06G0681	probable nuclear hormone receptor HR38 isoform X2	2.00 × 10^−8^	Down	C0-5D-VS-IBA-C1-5D
30	Gh_A06G1176	ethylene-responsive transcription factor ERF091-like	1.78 × 10^−54^	Down	C0-20D-VS-IBA-C1-20D
31	Gh_D08G1225	ethylene-responsive transcription factor ERF061-like	8.50 × 10^−49^	Ups	C0-5D-VS-IBA-C1-5D
32	Gh_A08G1503	ethylene-responsive transcription factor 5-like	8.27 × 10^−50^	Down	C0-20D-VS-IBA-C1-20D
33	Gh_A12G1021	dof zinc finger protein DOF3.7	5.10 × 10^−6^	Ups	C0-20D-VS-IBA-C1-20D
34	Gh_A06G1830	B-box zinc finger protein 21-like, partial	3.86 × 10^−6^	Down	C0-20D-VS-IBA-C1-20D
35	Gh_D11G1798	B-box zinc finger protein 18-like isoform X1	4.60 × 10^−6^	Ups	C0-5D-VS-IBA-C1-5D
36	Gh_A11G1640	B-box zinc finger protein 18-like isoform X1	4.61 × 10^−6^	Ups	C0-5D-VS-IBA-C1-5D

## Abbreviations

DEG	Differentially expressed gene
DGE	Digital gene expression
EC	Embryogenic callus
ERF	Ethylene response factor
GO	Gene Ontology
IAA	Indole acetic acid
IBA	Indole-3-butyric acid
KEGG	Kyoto Encyclopedia of Genes and Genomes
KT	Kinetin
MAPK	Mitogen-activated protein kinase
MS	Murashige and Skoog
NAA	Naphthalene acetic acid
NR	Non-redundant Protein Sequence Database in GenBank
RPKM	Reads Per Kilo bases per Million reads
RT-PCR	Reverse-transcription-polymerase chain reaction
SE	Somatic embryogenesis
*SERK*	Somatic embryogenesis receptor kinase
2, 4-D	2, 4-dichlorophenoxyacetic acid

## Appendix A

**Table A1:** Primers sequence for qRT-PCR.

Gene	Gene ID	Full Name	Primer Sequence (Forward/Reverse)	Tm (°C)	Product (bp)
CHIT1B	Gh_D06G0479	Basic endochitinase	AGCAGGAGAAGCAATAAAGG/ATGACACCGTATCCAGGGAC	56.6	201
CBL4	Gh_D11G0140	Calcineurin B-like protein 4	TCTTGCTGCTGAAACACCT/TCTAACGAACTCACCGAAC	53.4	235
PER73	Gh_D03G0246	Peroxidase 73	GCAACTATCCGCCTCTTCT/GCTTAACCCATCCAACCTC	54.3	291
CYP82A3	Gh_D05G2983	Cytochrome P450 82A3	CTTTGGTAGTGGTAGGAGGAG/GAGCAAGACGAGGTGAGAC	51.9	194
ARF4	Gh_D05G0755	Auxin response factor 4	CAACATGAATCAAGCACCCAA/GTTTCCTGATGTCGTCCAC	53	194
AUX22B	Gh_D13G1819	Auxin-induced protein 22B	GGCAACTCTTCGGAGCAAC/TATCCCACTAATTTCACCC	49.8	193
GhUB7	DQ116441	Ubiquitin 7	GAAGGCATTCCACCTGACCAAC/CTTGACCTTCTTCTTCTTGTGCTT	59	198
